# Dose‐dependent cranial irradiation associations with brain structures and neuropsychological outcomes in children with posterior fossa brain tumors

**DOI:** 10.1002/brb3.70019

**Published:** 2024-09-18

**Authors:** Mary Baron Nelson, Sharon H. O'Neil, Scarlet J. Cho, Sofia Dhanani, Jeffrey Tanedo, Brandon J. Shin, Jack Rodman, Arthur Olch, Kenneth Wong, Marvin D. Nelson, Jonathan Finlay, Natasha Lepore

**Affiliations:** ^1^ Department of Radiology Keck School of Medicine of USC Los Angeles California USA; ^2^ CIBORG Laboratory Children's Hospital Los Angeles Los Angeles California USA; ^3^ Department of Pediatrics Keck School of Medicine of USC Los Angeles California USA; ^4^ Neuropsychology Core The Saban Research Institute, Children's Hospital Los Angeles Los Angeles California USA; ^5^ Division of Neurology Children's Hospital Los Angeles Los Angeles California USA; ^6^ Department of Psychological Science, School of Social Ecology University of California Irvine Irvine California USA; ^7^ Division of Child Neurology, Department of Neurology Stanford University School of Medicine Stanford California USA; ^8^ Kansas City University College of Osteopathic Medicine Joplin Missouri USA; ^9^ Biostatistics, Epidemiology, and Research Design (BERD) Southern California Translational Science Institute Los Angeles California USA; ^10^ Department of Radiation Oncology, Keck School of Medicine of USC and Radiation Oncology Program Children's Hospital Los Angeles Los Angeles California USA; ^11^ Nationwide Children's Hospital Columbus Ohio USA

**Keywords:** cognitive functioning, cranial irradiation, magnetic resonance imaging, posterior fossa tumor

## Abstract

**Background:**

Posterior fossa irradiation with or without whole brain irradiation results in high doses of radiation to the thalamus, hippocampus, and putamen, structures critical to cognitive functioning. As a result, children with brain tumors treated with cranial irradiation (CRT) may experience significant cognitive late effects. We sought to determine the effect of radiation to those structures on neuropsychological outcome.

**Methods:**

Forty‐seven children with a history of posterior fossa tumor (17 treated with surgery; 11 with surgery and chemotherapy; and 19 with surgery, chemotherapy, and CRT) underwent neuroimaging and neuropsychological assessment at a mean of 4.8 years after treatment, along with 17 healthy sibling controls. The putamen, thalamus, and hippocampus were segmented on each participant's magnetic resonance imaging for diffusion indices and volumes, and in the radiation treatment group, radiation dose to each structure was calculated.

**Results:**

Performance on visuoconstruction and spatial learning and memory was lower in patient groups than controls. Volume of the thalamus, when controlling for age, was smaller in the patient group treated with CRT than other groups. Higher radiation doses to the putamen correlated with higher fractional anisotropy in that structure. Higher radiation dose to the hippocampus correlated with lower spatial learning, and higher dose to thalami and putamina to lower verbal and nonverbal reasoning.

**Conclusions:**

All children with posterior fossa tumors, regardless of treatment modality, had cognitive deficits compared to their sibling controls. Posterior fossa irradiation may affect thalamic volume and aspects of verbal and nonverbal cognitive functioning.

## Graphical abstract

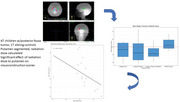

## 1 INTRODUCTION

Central nervous system tumors recently surpassed leukemia as the most common cancer in children aged 0–14 with an incidence of 5.83 per 100,000^−1^ and are now the leading cause of cancer mortality for children (Ostrom et al., 2020). Management of pediatric brain tumors, the majority of which occur in the posterior fossa, combines surgical resection, chemotherapy, targeted therapy or immunotherapy, and craniospinal or focal radiation therapy depending on the child's age at diagnosis, tumor location, pathology, and molecular and genetic subtype (Pollack et al., 2019). Long‐term effects of these tumors on cognition (Cheung et al., 2018; Robinson et al., 2013; Wagner et al., 2020), emotional function (Prasad et al., 2015), social adjustment (Schulte et al., 2018), and adaptive functioning (Panwala et al., 2019) are well‐documented and predominantly linked to cranial radiation therapy (CRT) (Nathan, 2007). Studies demonstrate a dose‐dependent effect of CRT on cognition (Toussaint et al., 2019); children who received higher doses had poorer long‐term neurocognitive outcomes (Grill et al., 1999; Merchant et al., 2014). Newer treatment techniques may contribute to less severe cognitive disability (Kahalley et al., 2019; Lassaletta et al., 2023; Levitch et al., 2022), but CRT‐related alterations in brain volume (Acharya et al., 2021; Rashid et al., 2017), and microstructure shown by diffusion tensor imaging (DTI) are common (Rueckriegel et al., 2015; Steen et al., 2001). Many studies compare patients to unrelated controls, include all tumor locations, and do not delineate effects of the tumor, surgical resection, or chemotherapy. In our previous study, we demonstrated the differential effects of surgical resection and chemotherapy on brain structure and neuropsychological outcomes in children with brain tumors who did not receive CRT, and showed that the addition of chemotherapy to surgical treatment carries its own independent neurotoxicity to brain microstructure and neuropsychological outcomes (Baron Nelson et al., 2021).

We focused on the thalamus, putamen, and hippocampus because when a high dose of focal photon beam irradiation is directed at the posterior fossa or cerebellum, as with medulloblastoma treatment, these structures, relevant to important neuropsychological domains (Bisecco et al., 2018; O'Shea et al., 2016; Riggs et al., 2014), receive significant irradiation. Functional magnetic resonance imaging (MRI) demonstrates brain structure activity when subjects are working on specific cognitive tasks (Baudou et al., 2024; Fama & Sullivan, 2015). The thalamus mediates executive function due to its extensive connectivity to the prefrontal cortex (Ouhaz et al., 2018), and thalamic and putamen volume predict attention and processing speed performance (Bisecco et al., 2018). Diffusion tensor imaging studies demonstrate the putamen has connections with the prefrontal cortex, primary somatosensory cortex, primary motor area, premotor cortex, thalamus, and cerebellum (Leh et al., 2010). Radiation dose to the hippocampus is inversely correlated to memory testing scores (Acharya et al., 2021; Gondi et al., 2010; Ma et al., 2017).

We aimed to determine differences in brain microstructure and neuropsychological functioning in children with posterior fossa tumors compared to healthy sibling controls and explored the relationship of radiation dose to the thalamus, putamen, and hippocampus to neuropsychological outcomes.

## 2 METHODS

The study, with a cross‐sectional and comparative design, was approved by the Institutional Review Board at Children's Hospital Los Angeles (CHLA‐14‐00334). Participants between 6 and 17 years of age underwent brain MRI with DTI and neuropsychological assessment. Children with posterior fossa tumors completed treatment at least 12 months prior to study with either surgery (Group 1), surgery and chemotherapy (Group 2), or surgery, chemotherapy, and CRT (Group 3). After identification of potential subjects from a brain tumor database and clinic schedules, parents were approached in clinic or contacted by mail or phone. Informed consent was obtained from parents and assent from children ≥7 years of age. Parents were invited to enroll healthy 6‐ to 17‐year‐old siblings of patients as controls. All participants were fluent in English, and their parents had to speak and read either English or Spanish.

Potential participants were excluded from the study for the presence of metal in the body precluding MRI, history of preterm birth, neurodevelopmental disability, or traumatic brain injury. Controls underwent MRI without sedation. Patients with a history of posterior fossa syndrome, recurrent tumor, or residual disease outside the posterior fossa were excluded.

All data were stored in REDCap v6.14.2 (Harris et al., 2019, 2009).

### 2.1 Imaging data and preprocessing

A 3.0T Philips Achieva scanner obtained three‐dimensional T1‐weighted images on participants. Voxel size was 1.0 × 1.0 × 1.0 mm^3^ with parameters: repetition time (TR), 9.9 ms; echo time (TE), 4.6 ms; 240 × 231 matrix; field of view (FOV), 24 cm. Diffusion weighted imaging (DWI) acquisition sequence parameters were: 70 axial slices (2 mm thick), FOV = 256 mm × 256 mm × 140 mm, TR/TE 8657/86 ms, no gap, with a 128 × 128 acquisition matrix, 28 gradient directions collected with *b*‐value = 1500 s mm^−2^.

Preprocessing, including linear registration and bias field correction, was done as described in our previous work (Baron Nelson et al., 2021).

T1‐weighted (T1w) registered images and DTI images were used for manual segmentation of the hippocampus, thalamus, and putamen in ITK‐Snap by co‐authors MN, SC, SD, and KH, and finalized by MN. Inter‐rater reliability was 0.97. Segmentations on T1w images were used to calculate structural volume, then overlaid onto DTI images to calculate mean fractional anisotropy (FA) and mean diffusivity (MD) in each structure. T1w images for Group 3 were manually registered via rotation and scaling to the patient's radiation planning. CT images and linear interpolation transformation were performed using ITK‐Snap to transform T1w images to CT space. Transformation coordinates for rotation and scaling from the T1w process were applied to the segmentations for the same patient to ensure consistency in the transformation process, and nearest neighbor interpolation was performed. To bring radiation dose‐fused CT images, which originally had half the number of slices of the anatomical CT, into the same space, they were resliced using linear interpolation in ITK‐Snap, without additional transformation. Transformed segmentations were overlaid on the modified radiation dose‐fused CT, and average radiation dose in Gray units was obtained for each individual structure.

### 2.2 Neuropsychological assessment

Participants completed measures to assess intellectual functioning, attention, executive functions, processing speed, learning and memory, and social‐emotional functioning. Assessments were performed by a board‐certified pediatric neuropsychologist or by doctoral trainees under her supervision.

### 2.3 Statistical methods

To determine differences in volume, FA, and MD in the thalamus, putamen, and hippocampus between the three treatment groups and the control group and differences in neuropsychological test scores, one‐way analysis of variance (ANOVA) or Kruskal–Wallis tests, depending on distribution, were used to evaluate overall differences in structural volume, mean FA/MD, and test scores. Post hoc pairwise comparisons with Bonferroni adjustment tested significant differences between groups. A sub‐analysis assessing association between neuropsychological test scores and demographic and treatment variables was done using *T*‐tests and Chi‐square tests, as appropriate.

To determine whether structural volume and FA/MD of the thalamus, putamen, and hippocampus were associated with radiation dose in Group 3, structural volume and mean radiation dose were determined for both left and right hemisphere for each structure. Total volume was then calculated by the sum of left and right hemisphere volumes for each, and radiation dose to the whole structure was represented by the mean of the dose to the right and left. Pearson or Spearman correlation, depending on distribution, assessed these associations. In Group 3, these correlations were used to assess associations between radiation dose to hippocampus and memory test scores, and between radiation dose to thalamus or putamen and general intelligence, executive functions, and processing speed. Linear regression determined how radiation dose affected structural volume, FA, and MD. Age at study was included as a covariate in the linear regression model. All tests were two‐sided and a *p*‐value of ≤.05 was considered statistically significant. Post hoc analyses were performed to determine whether right or left lateral mean values of structures (volume, CRT dose, FA, and MD) contributed significantly to correlations or mean differences. All analyses were done in R version 4.0.2.

## 3 RESULTS

### 3.1 Demographics

Demographic results are shown in Table 1.

**TABLE 1:** Demographics by treatment group.

Variable	*N*	Treatment group				*p*‐value
		Surgery only (1) (*n* = 17)	Surgery & chemo (2) (*n* = 11)	Surgery, chemo & radiation (3) (*n* = 19)	Healthy control (*n* = 17)	
Current age	64	10.0 (7.0) (6, 18)	13.0 (5.5) (7, 16)	12.0 (4.0) (6, 16)	11.0 (3.0) (7, 15)	.46
Age at diagnosis	47	5.1 (2.7) (1.4, 14.5)	2.3 (2.5) (1.0, 12.6)	7.6 (7.1) (0.7, 13.3)	——‐	.01*
**Time off treatment (years)**	47	4.5 (3.3) (1.0, 10.4)	8.1 (4.7) (1.2, 14.6)	3.1 (2.0) (1.0, 6.2)	——–	<.001*
**Diagnosis** **Medulloblastoma** **Pilocytic Astrocytoma** **Astrocytoma** **Ependymoma** **Glioneuronal tumor** **Choroid plexus** **Carcinoma** **Atypical teratoid** **Rhabdoid tumor**	47	0 14 (82%) 2 (12%) 0 1 (6%)	7 (64%) 2 (18%) 0 1 (9%) 1 (9%)	17 (89%) 0 0 0 2 (11%)	——‐	<.001*
**Race** White African American Asian Pacific Islander	59 1 3 1	15 (88.2%) 1 (5.9%) 1 (5.9%) 0	10 (90.9%) 0 1 (9.1%) 0	17 (89.5%) 0 1 (5.3%) 1 (5.3%)	17 (100%) 0 0 0	.85
**Ethnicity** Not Hispanic Hispanic/Latino Missing	31 32 1	7 (43.8%) 9 (56.3%) 1 (6%)	3 (27.3%) 8 (72.7%) 0	12 (63.2%) 7 (36.8%) 0	9 (52.9%) 8 (47.1%) 0	.27
**Family income** <$19,999 $20,000–39,999 $40,000–59,999 $60,000–79,999 $80,000–99,999 >$100,000 Missing	8 15 5 3 3 25 5	2 (14.3%) 4 (28.6%) 0 1 (7.1%) 0 7 (50.0%) 3 (18%)	1 (11.1%) 3 (33.3%) 0 1 (11.1%) 1 (11.1%) 3 (36.8%) 2 (18%)	4 (21.1%) 3 (15.8%) 3 (15.8%) 1 (5.3%) 1 (5.3%) 7 (36.8%) 0	1 (5.9%) 5 (29.4%) 2 (11.8%) 0 1 (5.9%) 8 (47.1%) 0	.85
**Mother's education** Less than 12 years High school diploma Some college Associate's degree Bachelor's degree Some graduate school Graduate degree Missing	17 13 3 1 9 3 14 4	5 (29.4%) 2 (11.8%) 1 (5.9%) 0 0 1 (5.9%) 5 (29.4%) 3 (17.6%)	5 (45.5%) 0 1 (9.1%) 1 (9.1%) 2 (18.2%) 1 (9.1%) 0 1 (9.1%)	1 (5.3%) 7 (36.8%) 1 (5.3%) 0 5 (26.3%) 1 (5.3%) 4 (21.1%) 0	6 (35.3%) 4 (23.5%) 0 0 2 (11.8%) 0 5 (29.4%) 0	.36
**Father's education** Less than 12 years High school diploma Some college Associate's degree Bachelor's degree Some graduate school Graduate degree Missing	11 7 15 2 9 0 15 5	2 (11.8%) 4 (23.5%) 3 (17.6%) 0 1 (5.9%) 0 3 (17.6%) 4 (23.5%)	4 (36.4%) 1 (9.1%) 1 (9.1%) 1 (9.1%) 2 (18.2%) 0 1 (9.1%) 1 (9.1%)	2 (10.5%) 2 (10.5%) 4 (21.1%) 1 (5.3%) 4 (21.1%) 0 6 (31.6%) 0	3 (17.6%) 0 7 (41.2%) 0 2 (11.8%) 0 5 (29.4%) 0	.46

*Note*: Numbers represent median (IQR) (min, max) for continuous variables and frequency (column percent) for categorical data.

Significant at *p = .05 (Fisher's exact).

Seven children in Group 3 (37%) were initially treated with high‐dose chemotherapy with autologous stem cell rescue as per Head Start protocols (Dhall et al., 2008; Gardner & Finlay, 2001) to avoid or reduce the dose/volume of cranial irradiation (Dhall et al., 2020), while the remaining children were treated as per Children's Oncology Group protocols. A comparison of chemotherapy agents given to children in Groups 2 and 3 is shown in Table 2.

**TABLE 2:** Chemotherapy treatment in Groups 2 and 3.

Chemotherapy agent	Children treated in Group 2 (%)	Children treated in Group 3 (%)
**Thiotepa**	82	37
**Etoposide**	64	37
**Carboplatin**	82	47
**Cisplatin**	73	95
**Cyclophosphamide**	82	100
**Vincristine**	100	100
**Temozolomide**	0	5
**Vinblastine**	0	5
**Methotrexate**	45	37
**Lomustine**	0	42

Seventeen of 19 children (89%) treated with CRT received whole brain irradiation in addition to a tumor bed or posterior fossa boost, while two received only focal radiation. Mean doses are shown in Table 3.

**TABLE 3:** Radiation doses for Group 3 participants.

	Mean dose (SD) [*n*]	Range
Whole brain	24.46 Gy [17]	18–36 Gy
Posterior fossa	40.35 Gy (15.17) [19]	19.8–55.8 Gy
Tumor bed	52.50 Gy (7.52) [18]	23.4–59.4 Gy
Hippocampus total mean dose Right hippocampus mean dose Left hippocampus mean dose	41.09 Gy (8.06) [16] 40.76 Gy (9.26) [16] 41.42 Gy (8.65) [16]	19.55–53.26 Gy 13.89–53.12 Gy 25.21–53.39 Gy
Thalamus total mean dose Right thalamus mean dose Left thalamus mean dose	36.12 Gy (9.39) [16] 36.34 Gy (8.99) [16] 35.01 Gy (8.39) [16]	11.68–53.25 Gy 10.24–50.41 Gy 13.11–50.25 Gy
Putamen total mean dose Right putamen mean dose Left putamen mean dose	32.37 Gy (7.77) [16] 33.13 Gy (8.66) [16] 31.60 Gy (8.07) [16]	10.7–45.73 Gy 10.23–46.83 Gy 11.1–26.09 Gy

Three participants in Group 3 received proton‐beam irradiation at another institution and were excluded from the radiation dose analyses because radiation planning images were not available. Each remaining participant was treated with intensity‐modulated radiation therapy (IMRT) using 6 MV X‐rays.

### 3.2 DTI‐ and MRI‐based volume comparison between groups

After adjusting for age, patients that received surgery, chemotherapy, and radiation had an overall thalamic volume that was, on average, 2253.84 units smaller compared to healthy controls (95% confidence interval [CI] = −3786.48 to −721.20; *p*‐value = .005). There was no significant difference in volumes of the hippocampus or putamen between groups.

Hippocampal FA was lower in patients than controls (*p* = .02). There was no difference in MD in these groups. We completed post hoc analyses for each right and left structure FA, MD, and volume to determine whether there were lateralized differences between the patient and control groups. Left (*p* = .01) but not right (*p* = .06) hippocampal FA was significantly lower in patients. Right (*p* = .02) and left (*p* = .002) thalamus were smaller in patients who were treated with surgery, chemotherapy, and CRT, with age as a covariate.

### 3.3 Radiation doses and DTI/volumes in Group 3

**FIGURE 1:**
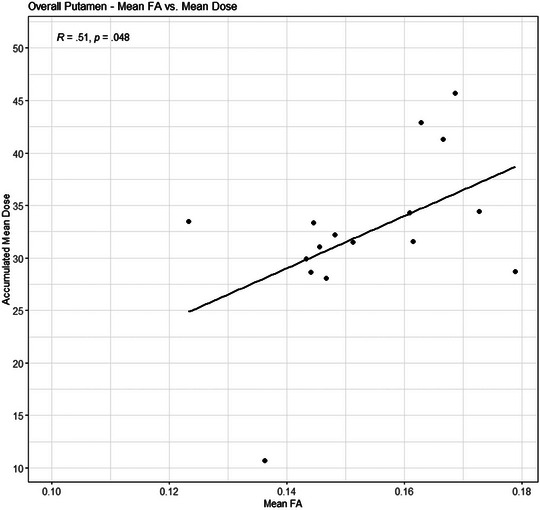
Fractional anisotropy (FA) in putamen increases as radiation dose increases.

In children treated with CRT, FA in the putamen increased as radiation dose increased (Figure 1), but radiation dose was not correlated to FA in the hippocampus or thalamus. Mean radiation dose to the thalamus was inversely correlated to MD in the putamen (*R* = −.585, *p* = .03), but not to MD in the thalamus (*R* = −.382, *p*‐value = .18).

Although mean thalamic volume was smallest in children in Group 3 while controlling for age, radiation dose was not correlated to thalamic volume (95% CI = −166.65 − 145.6; *p*‐value = .89).

### 3.4 Neuropsychological assessment

Results of neuropsychological testing are shown in Table 4.

**TABLE 4:** Mean neuropsychological assessment scores and percentage of subjects scoring at least 1 standard deviation (SD) below mean.

Domain/measure	Treatment group								
	Group 1 mean (SD) {range} [*n*]	Group 1 scoring ≥1 SD below mean	Group 2 mean (SD) {range} [*n*]	Group 2 scoring ≥1 SD below mean	Group 3 mean (SD) {range} [*n*]	Group 3 scoring ≥1 SD below mean	Healthy Control (HC) group mean (SD) {range} [*n*]	HC group scoring ≥1 SD below mean	*p*‐value (mean scores)
**Intellectual functioning** FSIQ‐4^35^	96.5^a^ (12.4) {78,116} [14]	21%	96.80^a^ (18.34) {74,140} [10]	20%	95.1^a^ (11.0) {73,114} [19]	16%	102.4^a^ (11.0) {87,122} [17]	0	.56
**Intellectual functioning** Block Design (Kim et al., 2008)	45.9^b^ (10.6) {28,58} [14]	29%	44.80^b^ (14.20) {29,73} [10]	60%	42.6^b^ (7.8) {30,58} [19]	47%	54.7^b^ (6.7) {44,67} [17]	0	**.01**
**Intellectual functioning** Matrix Reasoning (Kim et al., 2008)	48.9^b^ (8.8) {29,62} [14]	14%	50.1^b^ (11.0) {33,65} [10]	10%	48.0^b^ (10.1) {34,74} [19]	26%	52.4^b^ (7.3) {38,70} [17]	6%	.53
**Intellectual functioning** Vocabulary (Kim et al., 2008)	49.9^b^ (9.9) {34,64} [14]	21%	47.7^b^ (11.2) {31,71} [10]	30%	50.0^b^ (10.2) {29,69} [19]	21%	49.1^b^ (7.1) {37,63} [17]	12%	.93
**Intellectual functioning** Similarities (Kim et al., 2008)	48.2^b^ (5.2) {41,58} [14]	0/0	50.2^b^ (10.0) {37,77} [10]	10%	49.2^b^ (6.2) {38,60} [19]	11%	46.1^b^ (5.8) {38,61} [17]	12%	.45
**Processing speed** WISC/WAIS Coding (Boström et al., 2019)	8.36^c^ (3.03) {3,13} [14]	36%	8.78^c^ (4.89) {3,17} [9]	40%	7.47^c^ (2.20) {5,13} [19]	63%	9.93^c^ (2.90) {3,15} [17]	12%	.21
**Processing speed** Symbol Search (Boström et al., 2019)	9.07^c^ (3.22) {5,15} [14]	43%	7.89^c^ (3.06) {4,12}[9]	44%	7.84^c^ (1.98) {5,11} [19]	32%	10.36^c^ (2.71) {6,14} [17]	24%	.06
**Processing speed** Pattern Comparison (Kondziella et al., 2009)	84.7^a^ (23.0) {33,110} [12]	58%	77.6^a^ (18.75) {47,107} [10]	70%	72.37a (17.5) {32,99} [19]	74%	87.44^a^ (14.2) {60,108} [16]	50%	.09
**Attention** Digit Span Forward (Boström et al., 2019)	9.00^c^ (3.60) {4,16} [14]	36%	10.30^c^ (4.00) {4,16} [10]	30%	10.21^c^ (3.20) {4,17} [19]	16%	10.57^c^ (0.94) {4,12} [17]	6%	.98
**Learning and memory** List A trials 1‐5^38^	54.42^b^ (10.36) {40,66} [12]	0	49.20^b^ (12.63) {29,69} [10]	20%	51.39^b^ (10.02) {30,66} [18]	11%	53.92^b^ (6.05) {44,65} [17]	0	.52
**Learning and memory** List A long delay free recall (Zhao et al., 2016)	0.31^d^ (0.93) {−1.0,1.5} [13]	15%	0.25^d^ (0.95) {−1.5,1.5} [10]	10%	0.18^d^ (1.12) {−2.5,2.0} [19]	16%	0.29^d^ (0.84) {−1.0,2.0} [17]	6%	.96
**Learning and memory** MD content (Lundin et al., 2017)	8.54^c^ (3.57) {2,14} [13]	46%	8.80^c^ (4.78) {1,15} [10]	50%	10.37^c^ (2.43) {4,13} [19]	5%	11.71^c^ (2.73) {7,15} [17]	6%	**.04**
**Learning and memory** MD spatial (Lundin et al., 2017)	9.08^c^ (3.66) {4,15} [13]	38%	9.40^c^ (3.17) {4,14} [10]	20%	10.53^c^ (2.48) {6,14} [19]	11%	9.79^c^ (3.33) {6,14} [17]	12%	.28
**Learning and memory** MDD content (Lundin et al., 2017)	9.42^c^ (3.34) {5,14} [12]	42%	8.40^c^ (3.20) {5,13} [10]	50%	10.63^c^ (2.36) {5,13} [19]	16%	11.21^c^ (2.01) {8,14} [17]	0	**.04**
**Learning and memory** MDD spatial (Lundin et al., 2017)	9.42^c^ (3.45) {4,14} [12]	42%	7.90^c^ (3.67) {2,13} [10]	60%	9.89^c^ (3.07) {4,14} [19]	32%	11.00^c^ (2.35) {7,13} [17]	12%	**.05**
**Executive functions** Flanker (Kondziella et al., 2009)	82.22^a^ (11.81) {60,100} [13]	54%	82.90^a^ (12.91) {65,106} [10]	60%	78.05^a^ (6.37) {66,90} [19]	84%	85.57^a^ (10.25) {72,110} [17]	59%	.24
**Executive functions** Card Sort (Kondziella et al., 2009)	89.88^a^ (11.90) {60,102} [13]	38%	90.30^a^ (17.90) {70,126} [10]	50%	88.37^a^ (9.88) {73,117} [19]	47%	95.64^a^ (13.64) {71,120} [17]	29%	.58
**Executive functions** Digit Span Backward (Boström et al., 2019)	10.93^c^ (3.58) {6,19} [14]	14%	9.10^c^ (3.45) {3,14} [10]	30%	10.11^c^ (3.21) {5,17} [18]	28%	10.57^c^ (2.44) {6,15} [16]	12%	.59
**Executive functions** Spatial Span Backward (Boström et al., 2019)	11.42^c^ (3.23) {5,17} [12]	8%	10.60^c^ (3.63) {4,16} [10]	10%	9.95^c^ (2.53) {6,15} [19]	16%	10.86^c^ (3.11) {5,16} [17]	18%	.63
**Executive functions** List Sort Working Memory (Kondziella et al., 2009)	104.80^a^ (13.73) {89,134} [10]	0	97.90^a^ (21.51) {69,129} [10]	40%	98.20^a^ (17.46) {47,124} [19]	16%	98.60^a^ (10.22) {80,113} [15]	20%	.71
**Executive functions** BRIEF Behavioral Regulation Index (Kazda et al., 2014)	51.57^b^ (12.94) {37,78} [14]	14%^e^	54.10^b^ (13.33) {37,81} [10]	10%^e^	45.84^b^ (9.58) {35,79} [19]	5%^e^	45.21^b^ (8.12) {34,58} [17]	0^e^	.07
**Executive functions** BRIEF Metacognition Index (Kazda et al., 2014)	52.50^b^ (12.86) {37,78} [14]	29%^e^	53.20^b^ (13.89) {36,76} [10]	20%^e^	49.21^b^ (13.98) {33,85} [19]	11%^e^	46.43^b^ (8.42) {37,70} [17]	6%^e^	.46
**Behavior/mood** CBCL total problems	54.36^b^ (12.33) {35,70} [14]	21%^e^	53.8^b^ (11.22) {29,71} [10]	10%^e^	44.53^b^ (11.65) {27,70} [19]	5%^e^	42.07^b^ (11.57) {24,62} [17]	0^e^	**.02**
**Behavior/mood** CBCL internalizing problems (Abayomi, 1996)	55.00^b^ (10.81) {33,69} [14]	14%^e^	57.60^b^ (10.51) {39,70} [10]	30%^e^	49.21^b^ (11.12) {34,72} [19]	16%^e^	47.14^b^ (10.21) {33,68} [17]	6%^e^	**.04**
**Behavior/mood** CBCL externalizing problems (Abayomi, 1996)	52.36^b^ (14.37) {34,68} [14]	36%^e^	50.10^b^ (10.92) {34,71} [10]	20%^e^	41.26^b^ (9.42) {30,66} [19]	5%^e^	43.64^b^ (9.50) {33,62} [17]	0^e^	**.03**

^a^ Standard scores have a mean of 100, SD = 15.

^b^ *T*‐scores have a mean of 50, SD = 10.

^c^ Scaled scores have a mean of 10, SD = 3.

^d^ *Z*‐scores have a mean of 0, SD = 1.

^e^ Percentage of scores within clinically relevant range.

ANOVA tests revealed lower performance on visuoconstructional reasoning (Block Design) and spatial learning and memory (Memory for Designs) for patient groups than for controls. Increased internalizing and externalizing symptoms were reported by parents for children in treatment groups. Two of three processing speed measures approached significance for lower treatment group performance compared to controls.

To further explore results, we documented the percentage of subjects in each group scoring at least one standard deviation below the mean.

**FIGURE 2:**
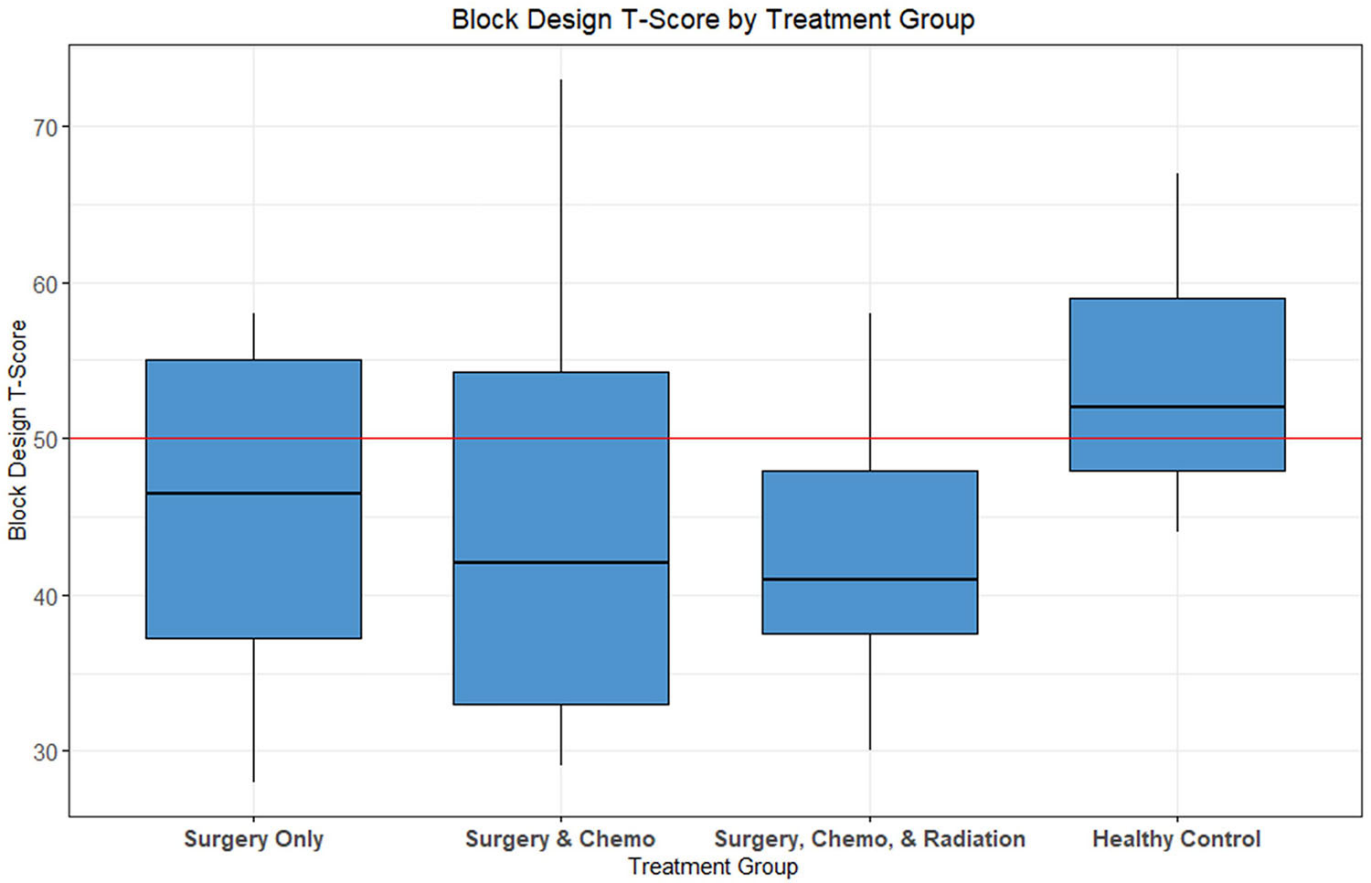
Boxplot of Block Design scores by group with the mean of *T* = 50 (SD = 10) in red.

Figure 2 displays the distribution of scores on Block Design in each patient group and the control group.

### 3.5 Radiation doses and neuropsychological scores in Group 3

**FIGURE 3:**
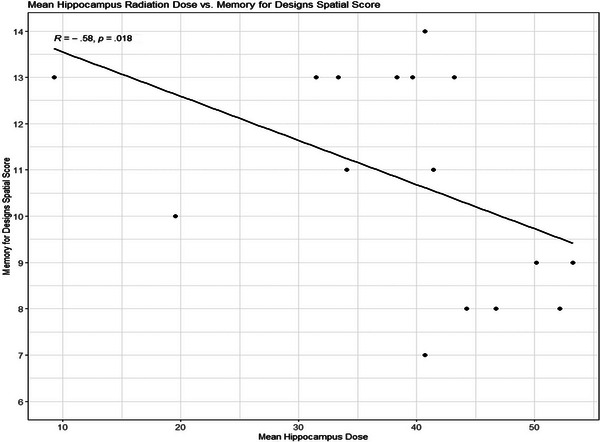
Relationship of mean hippocampal radiation dose to scores on memory for designs spatial.

**FIGURE 4:**
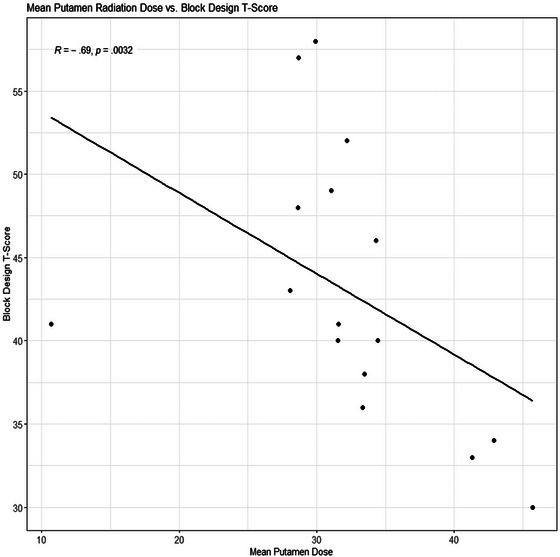
Relationship of mean putamen radiation dose to scores on Block Design.

**FIGURE 5:**
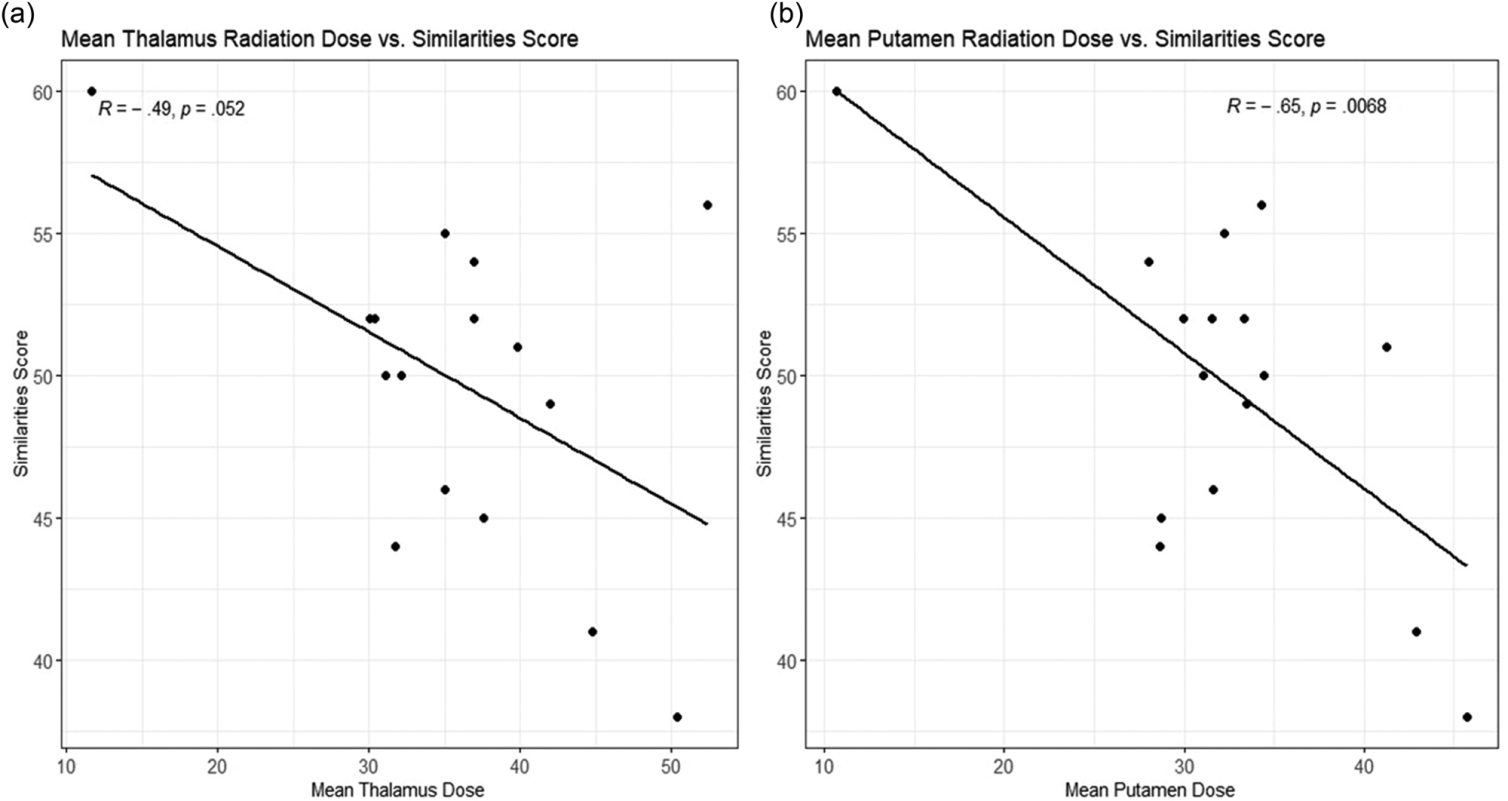
Relationship of mean thalamic (a) and putamen (b) radiation dose to scores on similarities.

Correlations between hippocampal radiation dose and Memory for Designs spatial learning, dose to putamen and Block Design, and dose to putamen and thalamus to Similarities verbal reasoning test are shown in Figures 3, 4, 5.

## 4 DISCUSSION

This study included 47 children with a history of posterior fossa tumor off‐treatment for at least 1 year with 17 of their siblings as healthy controls, reducing environmental factors in neuropsychological results. Aligned with demographics for the study location, 50% of the children identify as Hispanic/Latinx.

Children in Group 2 (surgery + chemotherapy) were younger at diagnosis than those in other treatment groups due to efforts to avoid CRT in young children by treating malignant brain tumors with myeloablative chemotherapy and stem cell transplant per Head Start protocols (Dhall et al., 2008; Gardner & Finlay, 2001). Therefore, many of these children were off treatment longer than those in Groups 1 (surgery) and 3 (surgery + chemotherapy + CRT). Tumor pathology differences between groups are inherent due to pathology‐based treatment. For example, there was a much higher prevalence of pilocytic astrocytoma in Group 1 and of medulloblastoma in the groups treated with the addition of CRT and/or chemotherapy.

Controlling for age, structural brain volumes between groups differed only in the thalamus. Thalamic volume was lower in children in Group 3 than in other treatment groups and controls. Radiation interferes with normal tissue growth through inflammation and oxidative stress (Kim et al., 2008). While the hippocampus received the highest mean radiation dose, followed by the thalamus and then the putamen, the thalamus has been shown to be sensitive to CRT, resulting in decreased neuronal and astrocytic density in rodent models (Boström et al., 2019), which could result in volume loss.

Another possible explanation for smaller thalamic volumes in Group 3 is that 84% of those children had hydrocephalus at diagnosis with 47% rated by the neuroradiologist as moderate or severe. Increased ventricular size and pressure may compress the adjacent thalamus causing injury resulting in volume loss (Kondziella et al., 2009) and alterations in DTI and spectroscopy indices (Lundin et al., 2017; Zhao et al., 2016). Hydrocephalus would also account for the finding that thalamic volume was not significantly correlated with CRT dose.

It may be that due to smaller structural size, hippocampal volume group differences did not reach statistical significance in this small sample. The feasibility of shielding the hippocampi during whole‐brain CRT is under investigation to determine whether doing so will ameliorate treatment‐related memory deficits (Kazda et al., 2014). Adults demonstrate significant decline in memory function after whole brain irradiation for brain metastases (Abayomi, 1996; Gondi et al., 2010) and hippocampal avoidance in adults may result in fewer memory deficits (Brodin et al., 2014; Zanirato Rambaldi et al., 2020). The hippocampus, because of the presence of neural progenitor cells, is particularly affected by chemotherapy (Dietrich et al., 2006; James et al., 2008; Mignone & Weber, 2006) and radiation (Amano et al., 2002; Monje et al., 2002). We found that all patients had lower FA in the hippocampus compared to controls.

In the putamen, FA increased as mean radiation dose to the structure increased. The significance of FA in gray matter is less clear than in white matter, since there is less directional organization in gray matter because of unmyelinated axon segments, neurons, and glial cells. A higher relative FA in gray matter may be a marker of injury. Elevated FA in the putamen and caudate nucleus was related to pressure from chronic subdural hematoma in older adults, correlating with the degree of intracranial pressure (Osuka et al., 2012). Higher FA in gray matter has also been associated with gliosis in a rodent model after traumatic brain injury (Bouix et al., 2013). In the putamen, MD decreased as radiation dose to the thalamus increased. The putamen is adjacent to the thalamus, separated by the internal capsule, and it is possible that the MD correlation was stronger in the gray matter of the putamen than in the mixed gray/white matter of the thalamus. Mean diffusivity reflects a reduction in intracellular barriers and may be higher in areas of axonal loss, edema, or chronic injury (Alexander et al., 2007; Cercignani et al., 2001; Kumar et al., 2006). Our previous work found significantly elevated MD in deep subcortical gray matter in children treated with intensive chemotherapy for brain tumors (Nelson et al., 2014). There are few studies on changes in MD after radiation therapy, but D. Wang et al. (2016) found decreased MD values shortly after brain irradiation, reflecting more acute injury. With FA and MD generally representing opposing characteristics of diffusion, it is likely our findings represent chronic radiation injury to the putamen after posterior fossa CRT.

Several key differences in neuropsychological outcomes between groups were indicated, though performance placed broadly within the average range for each group. Over 30 years ago, Mulhern et al. (1992) reported that children with brain tumors treated with CRT scored on average 12–14 points lower on tests of intellectual functioning than those who did not receive CRT. The subtle differences in outcome we found are likely related to improved CRT delivery with greater precision and lower dosing, and to the avoidance of CRT in younger children. Here, group differences were most strongly indicated for Block Design, a visuoconstructional reasoning task requiring participants to copy designs using blocks, and Memory for Designs, a spatial learning and memory task involving learning and recalling simple designs and their spatial locations. Examining the percentage of individuals performing one or more standard deviations (SD) below the mean helps describe these findings (see Table 4). Though different neuropsychological assessments make comparisons across studies difficult, our findings are consistent with prior research noting deficits in visual‐spatial memory in children treated for pilocytic astrocytoma (Aarsen et al., 2009; Liguoro et al., 2020) and medulloblastoma (Liguoro et al., 2020; Spiegler et al., 2003). In contrast to reported deficits in verbal learning and memory for children with posterior fossa tumors treated with surgery alone (Liguoro et al., 2020) or with CRT (Liguoro et al., 2020; Nagel et al., 2006), we did not find significant group differences. This corresponds to findings in pediatric pilocytic astrocytoma (Aarsen et al., 2009), in surgery with adjuvant chemotherapy (Baron Nelson et al., 2021), and in irradiation protocols (Spiegler et al., 2003).

Taken together, our findings suggest that the presence of a posterior fossa brain tumor and treatment, including surgery alone, affects visuospatial cognition including visuoconstruction and spatial learning and memory. Cerebellar damage can lead to impairments in these areas (Ahmadian et al., 2019). Further, visuoconstructional tasks are highly sensitive to CNS insult in general (Lezak et al., 2004), likely due to multiple discrete brain regions and networks involved, most notably the occipital, right parietal and frontal lobes, and associated subcortical structures.

Aggregate data indicated treatment groups did not perform lower than sibling controls in psychomotor processing speed, a key deficit noted in this population in the literature (Kahalley et al., 2013; Peterson et al., 2023; Rey‐Casserly & Diver, 2019), ostensibly due to white matter damage. Nevertheless, scores on two of three processing speed tests in our study approached group‐level significance. On the third task, two‐thirds (63%) of those in Group 3 scored ≥1 SD below the mean. Processing speed remains a key concern in this population, with our patient groups trending lower in this domain.

Regarding social‐emotional functioning, all group means were within the normal, non‐clinical range on standardized parent report (CBCL). Children in Group 2 had a higher degree of internalizing symptoms (i.e., anxiety, somatization, and depression). Consistent with recent literature (Mabbott et al., 2005; Y. Wang et al., 2022), participants who received CRT were not at increased risk for social‐emotional impairment. This suggests that the presence and treatment of a brain tumor is associated with poorer social‐emotional functioning for some children. Our findings of increased internalizing problems concur with reports from the Childhood Cancer Survivor Study that brain cancer survivors were more likely than siblings to report symptoms of depression (Zebrack et al., 2004) and somatization (Zeltzer et al., 2008). A recent meta‐analysis including studies with sibling, population, and convenience controls (Sharkey et al., 2020) reported pediatric brain tumor survivors had a higher level of depressive and anxious internalizing symptoms but not externalizing problems. Determining risk and resilience in social‐emotional outcomes is a relatively recent endeavor for this population (Brodin et al., 2014; Dietrich et al., 2006).

Higher radiation dose to the hippocampus was significantly related to lower performance on spatial learning but not recall. It is important to note that Group 3 participants all performed within normal limits, with as many children scoring above the mean as below, suggesting that strategies of postponing or reducing irradiation to the hippocampi may protect this region critical in both new learning and recall.

Higher radiation dose to the thalamus and putamen correlated with lower performance on verbal reasoning. Additionally, higher radiation dose to the putamen correlated with lower scores on visuoconstruction. The thalamus plays an active role in cognition (Lundin et al., 2017), connecting the cerebellum and subcortical regions to the cerebral cortex (Wiley et al., 2011). Interest in cognitive contributions of the basal ganglia has grown in recent decades.

Our purpose was to determine treatment‐based differences in structural brain volume and neuropsychological outcomes and the relationship between these variables for children treated for posterior fossa brain tumors. After controlling for as many variables as possible in a small study, we expected to see a more defined cumulative effect of additional therapies on the treatment continuum from surgery to the addition of chemotherapy and finally CRT, but our findings were not so clearly delineated. For example, children in Group 2 performed more poorly than Group 3 on spatial memory, and children in Group 1 often scored similarly to those who received more treatment. Although most children in Group 2 were treated with similar chemotherapy and doses, conditioning regimens varied slightly. There was more variability in chemotherapy given when comparing Groups 2 and 3. Volume and microstructure of the hippocampus, putamen, and thalamus, all close to the posterior fossa radiation field, were not uniformly affected by radiation.

In recent decades, radiation treatment techniques have greatly improved. IMRT improves the distribution of radiation dose to the targeted tumor bed while minimizing dose to surrounding tissue, contributing to less severe late effects such as hearing loss and neuropsychological deficits. Irradiating the brain with proton beams significantly decreases the exit dose of radiation (Scaringi et al., 2018) and may further minimize cognitive deficits.

Given that neuropsychological outcomes for patient groups in this study were within the average range overall, and in contrast to outcomes reported in previous decades (Mulhern et al., 1992), advances in treatment have had a significantly positive impact. Additionally, our results using healthy sibling controls indicate demographic variables likely have a larger impact on outcomes than is captured when using population norms or unrelated controls. Nevertheless, despite generally encouraging results, it is critical to look beyond group data, as individual variability is high, with some scores well below and well above average in all groups. As such, continued examination of risk and resilience factors is indicated.

A limitation of this study is the small sample size, common in single‐site pediatric brain tumor studies. Age at diagnosis and time off treatment, factors that may influence outcome, were variable. Pediatric brain tumor research consortiums are working to provide improved statistical power to define treatment variables related to outcomes. Another limitation is the use of DTI to draw inferences about alterations in the microstructural integrity of the brain. The reliability of FA measures in white matter is confounded by crossing fibers (Figley et al., 2022), and there are few studies on the interpretation of DTI indices in gray matter.

## 5 CONCLUSION

Our findings suggest that posterior fossa radiation may lead to decreased thalamic volume and injury to the putamen as measured by FA. Additionally, higher radiation doses to the hippocampus were significantly related to lower performance on spatial learning, and higher radiation doses to the thalamus and putamen correlated with lower performance on verbal reasoning. The presence of a posterior fossa brain tumor and treatment, including surgery alone, affects visuospatial cognition, and processing speed remains a concern in this population. Future studies with larger samples and advances in neuroimaging and machine learning may allow determination of lateralized impacts of irradiation to specific structures in the developing brain to inform treatment planning.

## AUTHOR CONTRIBUTIONS

**Mary Baron Nelson**: Conceptualization; investigation; funding acquisition; writing—original draft; methodology; validation; writing—review and editing; visualization; formal analysis; project administration; supervision. **Sharon H**. **O'Neil**: Investigation; writing—original draft; methodology; writing—review and editing; supervision. **Scarlet J. Cho**: Writing—original draft; writing—review and editing; visualization; formal analysis. **Sofia Dhanani**: Writing—original draft; validation; visualization; formal analysis; writing—review and editing. **Jeffrey Tanedo**: Investigation; methodology; visualization; validation; software; writing—review and editing; project administration. **Brandon J. Shin**: Writing—review and editing; visualization; methodology. **Jack Rodman**: Formal analysis; writing—review and editing. **Arthur Olch**: Methodology; validation; writing—review and editing. **Kenneth Wong**: Methodology; validation; writing—review and editing. **Marvin D Nelson Jr**: Visualization; resources. **Jonathan Finlay**: Conceptualization; writing—review and editing. **Natasha Lepore**: Supervision; conceptualization; methodology; writing—review and editing; software.

## CONFLICT OF INTEREST STATEMENT

The authors declare no conflicts of interest.

### PEER REVIEW

The peer review history for this article is available at https://publons.com/publon/10.1002/brb3.70019

## Data Availability

The data that support the findings of this study are available on request from the corresponding author. The data are not publicly available due to privacy or ethical restrictions.
